# Effects of ontogeny and oiling on the thermal function of southern sea otter (*Enhydra lutris nereis*) fur

**DOI:** 10.1093/conphys/coad095

**Published:** 2023-12-14

**Authors:** Kate Riordan, Nicole M Thometz, Francesca I Batac, Teri E Nicholson, Heather E M Liwanag

**Affiliations:** Department of Biological Sciences, California Polytechnic State University San Luis Obispo, 1 Grand Ave, San Luis Obispo, CA 93407, USA; Department of Biology, University of San Francisco, 2130 Fulton Street, San Francisco, CA 94117, USA; California Department of Fish and Wildlife, Marine Wildlife Veterinary Care and Research Center, 151 McAllister Way, Santa Cruz, CA 95060, USA; Monterey Bay Aquarium, 886 Cannery Row, Monterey, CA 93940, USA; Department of Biological Sciences, California Polytechnic State University San Luis Obispo, 1 Grand Ave, San Luis Obispo, CA 93407, USA

**Keywords:** Development, insulation, lanugo, pelage, thermal conductivity, thermal resistance

## Abstract

During the evolution of most marine mammals, fur as an insulator has been replaced with more buoyant, energy storing and streamlining blubber. By contrast, the sea otter (*Enhydra lutris*) relies on insulation from its dense, air-trapping pelage, which differs morphologically between natal and adult stages. In this study, we investigated the ontogenetic changes in thermal function of southern sea otter (*Enhydra lutris nereis*) pelts in air, in water, and when saturated with crude oil. Pelt thermal conductivity, thickness, and thermal resistance were measured for six age classes: neonate (<1 month), small pup (1–2 months), large pup (3–5 months), juvenile (6 months–1 year), subadult (1–3 years), and adult (4–9 years). Thermal conductivity was significantly higher for pelts in air than in water, with oiled pelts exhibiting the highest values (*P* < 0.001). Oiled pelts had the lowest thermal resistance, which suggests that regardless of age, all sea otters are vulnerable to the effects of oiling (*P* < 0.001). To scale up our laboratory findings, we used a volume-specific geometric model of conductive heat transfer for a simplified sea otter body, representing all tested age classes and treatments. Neonates, small pups, and large pups are more vulnerable to the effects of oiling compared with older age classes (*P* < 0.0001) due to a higher surface area-to-volume ratio. These results are consistent with the known thermal conductance values for adult sea otter pelts, yet this is the first time such thermal differences have been demonstrated in young otters. Overall, body size and age play a more important role in the thermal abilities of sea otters than previously thought.

## Teaser

We examined thermal function of sea otter pelts across ontogeny and when oiled. We found that younger sea otters are more vulnerable to heat loss after oiling, due to their small body size and lower hair density compared to older age classes with adult-like pelage.

## Introduction

The southern sea otter (*Enhydra lutris nereis*) population has increased significantly since 1938, when a small raft of 30–50 otters was first spotted in Big Sur, California, after this subspecies was presumed extinct (Bolin, 1938). Following conservation efforts by government agencies, non-profit organizations, and public stewardship, sea otters have expanded their range along the central California coastline, where the population has increased >50-fold (Hatfield *et al.*, 2019)*.* Despite sea otter conservation successes in California, any substantial population increase is still limited by their geographic constraints, overall slow growth rate, and significant mortality threats from white sharks, pathogen pollution, and toxic algal blooms (Miller *et al.*, 2020; USFWS, 2023). Another substantial threat to southern sea otter recovery is its vulnerability to a major oil spill event, one of the main reasons this population’s status was listed as threatened, as defined by the federal Endangered Species Act (Greenwalt, 1979; Van Blaricom and Jameson, 1982).

Sea otters are unique because they are endothermic homeotherms living in cold water (1–16°C), where they are constantly thermally challenged (Costa and Kooyman, 1984; Riedman and Estes, 1990). Seawater conducts heat 25 times faster than air at the same temperature, and a high surface area-to-volume ratio (SA:V) puts sea otters at risk of being thermally stressed in the ocean, including the cold waters of the California current (Costa and Kooyman, 1982). In response, sea otters evolved morphological adaptations within their pelage that enable their fur to function as an effective insulator in water (Liwanag *et al.*, 2012*a*, 2012b). Fur functions as an insulator by trapping air between the skin and the surrounding environment (Tregear, 1965; Korhonen and Harri, 1986; Reynolds, 1993; Russell and Tumlison, 1996). Sea otters have the densest fur of any mammal, and this air layer supplies 70% of the overall thermal insulation (Kenyon, 1969; Tarasoff, 1974; Williams *et al.*, 1988; Williams *et al.*, 1992). To maintain the insulative properties of their pelage, sea otters groom frequently (Reynolds and Rommel, 1999) to clean the fur, maintain its loft and air layer, and redistribute the natural oils over the skin and hairs (Williams *et al.*, 1988; Williams and Davis, 1995; Yochem and Stewart, 2009).

The unique structure and function of their fur makes sea otters particularly vulnerable to the effects of oil spills. When the fur becomes oiled, it is no longer able to trap air effectively, and the pelt loses its water-repelling qualities (Costa and Kooyman, 1982; Jessup *et al.*, 2012). Previous studies investigated the thermal conductance of adult sea otter pelts and found that oiling of sea otter fur causes a 70% decrease in thermal insulation, potentially leading to hypothermia (Davis *et al.*, 1988; Williams *et al.*, 1988). Despite a wealth of knowledge regarding the functional morphology of adult sea otter pelage and the negative effects of oiling, until recently very little was known about the characteristics of sea otter natal pelage, or the pelage of other age classes (juveniles and subadults).

Sea otters are born with a natal pelage, which they gradually molt into a more adult-like coat at ~13 weeks of age (Payne and Jameson, 1984; Riordan *et al.*, 2023). Recent research has described the morphological differences between the natal pelage and the adult pelage (Riordan *et al.*, 2023). Natal pelage-bearing age classes have longer guard hairs that increase pelt thickness (or loft), but their hair density is as low as one-fourth that of adult pelts (Riordan *et al.*, 2023). Given that these differences in pelt morphology could affect pelt thermal function, we hypothesized that sea otters with natal pelage could be more vulnerable to heat loss, particularly in water and when oiled. The aims of this study were to: (1) compare the thermal function of sea otter fur across ontogeny, in air and in water, (2) examine the effects of oiling on the thermal function of sea otter pelts across ontogeny, and (3) calculate the scaled volumetric heat loss for sea otters across ontogeny in various treatment conditions (in air, in water, oiled). Here, we provide measures of sea otter thermal function and pelt thickness, describe whole-animal heat loss, examine the potential drivers for the differences in insulation effectiveness across age classes, and determine whether certain age classes of sea otters are more vulnerable to the effects of oiling.

## Materials and Methods

### Sample collection

In collaboration with California Department of Fish and Wildlife (CDFW), sea otter pelts were collected from San Luis Obispo, Monterey, and Santa Cruz county strandings of animals that died in the wild or during rehabilitation efforts. In accordance with Section 109(h) of the U.S. Marine Mammal Protection Act (MMPA), and the U.S. Fish and Wildlife Service’s regulations implementing the MMPA at 50 CFR 18.22(a) and the U.S. Endangered Species Act at 50 CFR 17.21(c)(3), the samples used to complete this work were collected from fresh, necropsied sea otter carcasses recovered from the wild by an official or employee of CDFW in the course of their duties. Only pelts considered fresh and in good condition (i.e., not matted or decayed) were used for this study. The original 24 × 20 cm pelt swatches were collected from the back (dorsal) region of the animal. The samples were packaged in three layers of plastic food wrap, flattened (not folded), stored in 2-gallon freezer bags, and frozen (−20 to −16°C) until analysis in the various experiments. A total of 44 samples represented six age classes, including neonate pups (<1 month, N = 9) and small pups (1–2 months, N = 5) with the natal pelage, and large pups (3–5 months, N = 5), juveniles (6 months–1 year, N = 6), subadults (1–3 years, N = 10), and adults (4–9 years, N = 9) with a mature pelage type; sample sizes varied depending on the experimental condition (detailed below). No pelt samples from aged adult sea otters (≥10 years) were used in this study. Sea otter pelts were categorized into age classes by CDFW employees based on well-established sea otter stranding age estimation protocols that use total body length and tooth development data as identifiers (Nicholson *et al.*, 2020).

### Thermal function

To evaluate thermal function, we measured thermal conductivity (*k_i_*) and pelt thickness (*L_i_*), and we then calculated thermal resistance (*R*) for each pelt. Thermal conductivity (W·m^−1^·°C^−1^) is a material property that describes how easily heat moves through a particular substance, independent of thickness (Kvadsheim *et al.*, 1994). Thermal resistance (m^2^·°C·W^−1^), also called thermal insulance, describes how well a material resists the flow of heat, and it incorporates both the thermal conductivity and the thickness of the material. Thermal resistance is the inverse of thermal conductance (*C*), or heat transfer (Scholander *et al.*, 1950). Note that thermal conductivity is sometimes confused with thermal conductance, but the latter varies with the thickness of the material whereas the former is independent of thickness (Kvadsheim *et al.*, 1994). Because of this, we used thermal conductivity to compare the efficiency of the pelts, and we calculated thermal resistance to compare their overall effectiveness.

We measured thermal conductivity of each pelt in air, in water, and after application of crude oil to the fur, using the standard material method (Kvadsheim and Aarseth, 2002; Liwanag *et al.*, 2012*a*, 2012b; Sharma and Liwanag, 2017). Pelt samples were trimmed into 15 × 15 cm squares from the original dorsum pelt swatches. We washed each pelt in cold running water to remove any sand or dirt. We gently dabbed the fur with paper towels to help dry the pelt samples. We then used a hair dryer (Trezero^®^ 2200 W ceramic tourmaline blow dryer) on the cool setting to thoroughly dry the pelt, as it has been found that blow drying will help restore the insulating air layer in the pelage (Williams and Davis, 1995). To reduce the effects of freeze–thaw cycles on each pelt, we performed most in-air, in-water, and crude oil trials on the same day. For in-air trials, all 44 sea otter pelts were included: neonates (N = 9), small pups (N = 5), large pups (N = 5), juveniles (N = 6), subadults (N = 10), and adults (N = 9). Because some pelts used for in-water or oiled trials did not successfully maintain an air layer upon submergence (based on visual inspection), we had varying sample sizes for these trials. A total of 35 sea otter pelts were used for in-water trials: neonates (N = 8), small pups (N = 5), large pups (N = 5), juveniles (N = 5), subadults (N = 7), and adults (N = 5). A total of 35 sea otter pelts were used for oiled trials: neonates (N = 7), small pups (N = 5), large pups (N = 5), juveniles (N = 5), subadults (N = 8), and adults (N = 5).

To determine pelt thickness (*L_i_*), we took measurements of the skin and the fur loft to the nearest 0.01 mm from the right, left, and posterior sides of the pelt (three measurements on each side, nine total) using digital calipers (Absolute Digimatic Caliper Series 500, Mitutoyo^©^, Aurora, IL, USA). Mean pelt thickness values were used in calculations. We calculated dry pelt thickness as the skin thickness plus the fur loft (to the top of the guard hairs). To calculate wet pelt thickness for in-water and oiled trials, we measured fur loft using a blunt probe and a ruler while the pelt was set up in the apparatus with water on top of the air layer; this ensured we recorded the true thickness of the pelt during submergence. We estimated wet pelt thickness (for both in-water and oiled trials) by summing skin thickness (measured outside the apparatus) with the fur loft measured in the apparatus.

To collect thermal conductivity measurements, we used a heat flux chamber with a highly insulated lower compartment and chilled upper compartment, described in Pearson *et al.* (2014). The lower insulated compartment contained a sealed aluminum box heat source with heated water to mimic mammalian body temperature (37°C), maintained with a circulating water bath (model SD07R-20; PolyScience, Niles, IL, USA). The chilled upper compartment contained ice packs to produce a consistent thermal gradient in the apparatus. We used an elastomer (Plastisol vinyl; Carolina Biological Supply, Burlington, NC, USA) as the standard material (*k_standard_* = 0.099231361 ± 0.01459027 W·m^−1^·°C^−1^, *L_standard_* = 0.00790478 ± 0.00014422 m). During trials, we placed the standard material on top of the heat source and positioned each pelt sample on top of the standard material (Pearson *et al.*, 2014). We arranged copper-constantin (Type T) thermocouples (Physitemp Instruments, Inc., Clifton, NJ, USA) to measure temperature at the different interfaces. We placed three thermocouples between the surface of the heat source and the standard material, three thermocouples between the standard material and the pelt sample, and three thermocouples directly on top of the fur (Pearson *et al.*, 2014). Additionally, we placed two thermocouples on the surface of the skin, at the base of the fur. To measure the heat flux through the sample, we placed a heat flux disc (Thermonetics Inc., San Diego, CA, USA) between the pelt and the standard material and another heat flux disc on top of the pelt. We recorded the outputs for all thermocouples every 6 s onto a desktop computer, using a Fluke Hydra data logger (model 2625A; Fluke Inc., Everett, WA, USA). Trials proceeded for a minimum of 2 h to confirm that the apparatus reached a steady equilibrium state, and data were analysed from the final 30 min of each trial.

Using the Fourier equation, we calculated thermal conductivity across the pelt (Kreith, 1958):


(1) \begin{equation*} H={k}_i\bullet A\bullet \Delta T\bullet {L}_i^{-1} \end{equation*}

where *H* is heat transfer (J·s^−1^), *k_i_* is thermal conductivity (W·m^−1^·°C^−1^), *A* is area through which the heat is dissipating (m^2^), *ΔT* is the temperature differential (°C) across the pelt, and *L_i_* is pelt thickness (m), or the sum of skin thickness and hair loft, for a given treatment (in-air, in-water, oiled). Heat transfer was assumed to be equal across the standard material and pelt sample, and the equations were set equal to solve for pelt thermal conductivity (Scholander *et al.*, 1942; Scholander *et al.*, 1950; Hart *et al.*, 1959; Bryden, 1964; Worthy, 1991; Kvadsheim *et al.*, 1994). To account for changes in the insulation of the fur with development, we calculated thermal resistance (*R*; m^2^·°C·W^−1^) for each pelt using the equation:


(2) \begin{equation*} R={L}_i\bullet {k}_i^{-1} \end{equation*}

For in-water trials, we gently poured 40 ml of fresh water at 10°C on top of the pelt, and then placed three thermocouples just on top of the visible, silvery air layer. We used fresh water to ensure consistency in the thermal conductivity of the water for comparison across samples; thermal conductivity is inversely related to the salinity of water (IAPWS, 2015). For these trials, we only used the heat flux disc between the pelt and the standard material. We did not place a heat flux disc on top of the pelt for in-water and oiled treatments to avoid disturbing the air layer on top of the pelt. For crude oil trials, we placed plastic food wrap and sealant (Loctite^®^ clear silicone waterproof sealant, Westlake, OH, USA) underneath the pelt to ensure no crude oil seeped onto the thermocouples beneath (Pearson *et al.*, 2019); we cut out a 5 × 5 cm square in the plastic food wrap to ensure the thermocouples were in contact with the underside of the skin. To mimic oiling, we applied 10 ml of Scott Well unrefined crude oil (Texas Raw Crude^©^, Midland, TX, USA) at 25°C to the pelt using a syringe and gently massaged the oil into the fur for 30 s, similar to the grooming movements performed by a sea otter, ensuring the oil was evenly distributed across the pelt sample. Next, we placed 40–60 ml of water at 10°C on top of the oiled fur using a plastic transfer pipette. We placed three thermocouples on top of the wet, flattened fur, similar to the in-water treatment.

Once an oiled trial was completed, we washed the pelt using Dawn^®^ dishwashing detergent (Dawn^®^ Ultra Dishwashing Liquid, Proctor & Gamble, Cincinnati, OH, USA). All crude oil hazardous waste was collected and properly disposed using PPE (gloves, goggles, facial coverings). We applied Dawn^®^ to the oiled pelt using a syringe, and we gently massaged small amounts of the detergent into the pelt. We then used cold running water to remove the detergent and repeated adding small amounts of Dawn^®^ at a time, followed by rinsing. Time spent washing and the amount of Dawn^®^ needed to wash the pelt were recorded. Washed pelts were considered clean when no oil or soap residue was visible and the water coming off the pelt was clear. We conducted additional visual inspections of the pelt after the pelt was patted dry with paper towels to ensure all oil and soap were removed.

### Heat loss model

**Figure 1:**
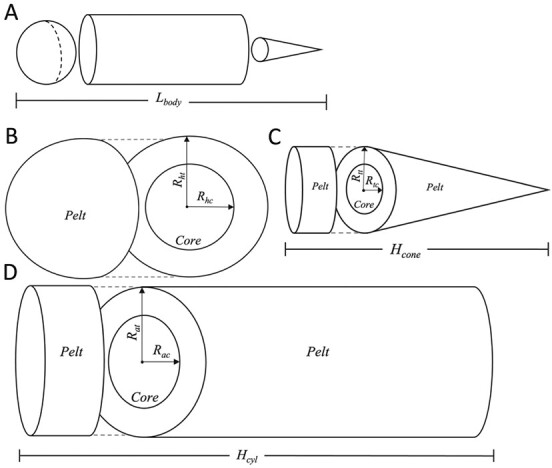
Geometric representations of sea otter body shape used to model heat loss across ontogeny. All of the models simulate a body core surrounded by a pelt layer. A) The three shapes (sphere, cylinder, cone) used to estimate heat loss, with total body length (*L_body_*). B) Spheroid model used to represent the head of the otter, as described in Eq. 3. C) Conical model of heat loss used to represent the tail, as described in Eq. 5. D) Cylindrical model of heat loss used to represent the trunk, as described in Eq. 4.

To scale our laboratory-based thermal conductivity findings to an entire animal, we estimated whole-body conductive heat loss through the pelt in air and in water following the methods in Pearson *et al.* (2019). We calculated total conductive heat transfer (*Heat_tot_*; W/m^3^) for sea otters as heat loss through a layer of insulation surrounding a body divided into three parts: head (sphere), trunk (cylinder), and tail (cone). We followed Eq. 27 from Mathewson and Porter (2013) and modelled heat flux (*Q*; W) for each body region (Fig. 1; see Supplementary Table S1 and Table S2). The heat flux of a sphere (*Q_sphere_*; W) was calculated as:


(3) \begin{equation*} {Q}_{sphere}=\frac{T_{MB}-{T}_A}{\frac{1}{4}\pi {k}_i\left[\frac{1}{R_{hc}}-\frac{1}{R_{ht}}\right]} \end{equation*}

where *T_MB_* is the temperature at the muscle-skin interface (°C), *T_A_* is the ambient temperature (set to 16°C for air and 13°C in water), *k_i_* is the thermal conductivity (W·m^−1^·°C^−1^) for a given treatment (in-air, in-water, oiled), *R_hc_* is the head core radius (m) and *R_ht_* is the total head radius (m), including separate pelt thickness for each treatment (*L_i_*; m). To account for known differences in pelt thickness along the body, we multiplied the pelt thickness by three-fourths to account for the decrease in pelt thickness on the head and tail. We used the subcutaneous temperature (*T_sq_*) value (36.5°C) from Costa and Kooyman (1982) as *T_MB_*. We averaged head girth values from data taken during routine CDFW live captures of adult (N = 10), subadult (N = 10), and large pup (N = 1) sea otters collected in November 2022. For the neonate and small pup age classes, we estimated the head girth by subtracting the difference found between juvenile and large pup age classes. We used the original head radius from the head girth values to estimate *R_hc_* and *R_ht_*. To calculate *R_hc_*, we used the head radius and subtracted the wet pelt thickness. We estimated all core radius values because the hair loft was flattened during girth data collection, and we subtracted the wet pelt thickness from each shapes’ core radius. The heat flux of a cylinder (*Q_cyl_*; W) was calculated as:


(4) \begin{equation*} {Q}_{cyl}=2\pi \bullet {H}_{cyl}\bullet {k}_i\left[\frac{T_{MB}-{T}_A}{\mathit{\ln}\left(\frac{R_{ac}}{R_{at}}\right)}\right] \end{equation*}

where *H_cyl_* is the cylinder height (m) or trunk length, *R_ac_* is the axillary core radius (m), and *R_at_* is the total axillary radius (m), which includes pelt thickness. To calculate *H_cyl_*, we used the total body length (*L_body_*; m) and subtracted cone height (*H_cone_*; m) and head diameter. We used *L_body_* and tail length values from the animals sampled for pelts in the present study, from CDFW live sea otter captures, and live neonate (N = 22), small pup (N = 21), and large pup (N = 12) rehabilitation patients from Monterey Bay Aquarium’s (MBA) Sea Otter Program. To calculate *R_ac_*, we used axillary girth values from CDFW live sea otter captures and live sea otter data collected from MBA’s Sea Otter Program patients. The heat flux of a cone (*Q_cone_*; W) was calculated as:


(5) \begin{equation*} {Q}_{cone}=\left[\frac{{\pi k}_i\left({T}_{MB}-{T}_A\right)}{H_{cone}}\right]\left({R}_{tc}\bullet {R}_{tt}\right) \end{equation*}

where *H_cone_* is the cone height (m) or tail length, *R_tc_* is the tail core radius (m), and *R_tt_* is the total tail radius (m), which includes pelt thickness. To calculate *R_tc_*, we determined a ratio of the known axillary and tail girth from an adult sea otter, then applied this relationship to estimate *R_tc_* for other age classes. For *H_cone_*, we used tail length values collected from the present study pelts. If any values were missing, we used the average value of that parameter by age class. To calculate the volume of each shape, we used the known volume equations of a sphere, cylinder and cone (Supplementary Table S1). To calculate the total heat loss for each treatment, we summed all the heat flux values from each shape and divided by the sum of their volumes (see Supplementary Material).

### Statistical analyses

All analyses were performed in R version 4.2.0 (R Core Team, 2022). A *P-*value < 0.05 was considered significant for all analyses. Thermal conductivity, thermal resistance, pelt thickness, and heat transfer were compared using models from the *lmerTest* package in R (Kuznetsova *et al.*, 2017). The models included treatments (in-air, in-water, oiled) and age classes as main effects, along with all possible interactions, and sea otter sample ID as a random effect. To make pairwise comparisons, we computed estimated marginal means for treatment and age class combinations in our models using the *emmeans* package in R (Lenth, 2018). To compare for differences across age classes related to the amount of Dawn^®^ needed to clean each pelt and the amount of cleaning time to reverse the crude oil treatment, we used a one-way ANOVA followed by a Tukey honest significant difference test. To investigate the relationship between sea otter hair density and thermal conductivity, we applied a linear regression model. Hair density values were obtained from a previous study of the same pelt samples, and values were matched by individual (Riordan *et al.*, 2023).

## Results

### Thermal function

#### Thermal conductivity

**Figure 2:**
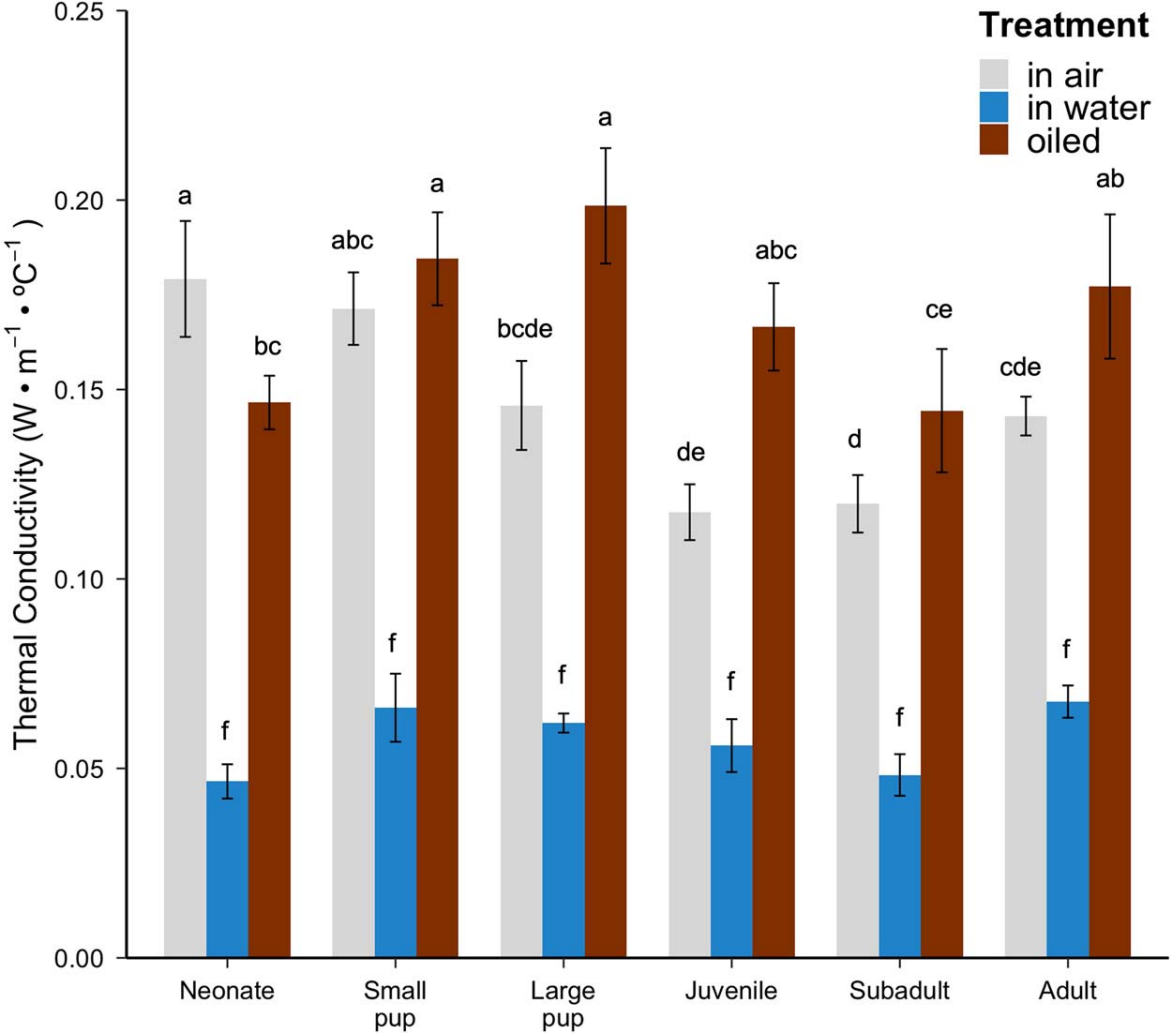
Thermal conductivity (W·m^−1^·°C^−1^) of sea otter pelts across ontogeny in air, in water and after oiling with crude oil. Heights of the bars and lines indicate means and standard errors for associated treatment and age class: neonate (N = 9 in air, N = 8 in water, N = 7 oiled), small pup (N = 5 for all treatments), large pup (N = 5 for all treatments), juvenile (N = 6 in air, N = 5 in water, N = 5 oiled), subadult (N = 10 in air, N = 7 in water, N = 8 oiled), and adult (N = 9 in air, N = 5 in water, N = 5 oiled). Different letters above the bars indicate statistically significant differences among means.

**Figure 3:**
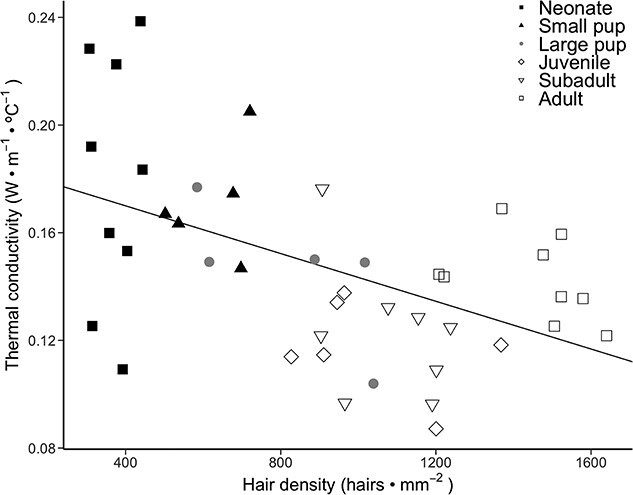
Correlation between hair density (hairs·mm^−2^) and thermal conductivity (W·m^−1^·°C^−1^) of sea otter pelts in air. Each sample is represented by a single symbol, and different symbols and colors represent different age classes. Line represents the best-fit linear regression.

Sea otter pelt thermal conductivity differed significantly across age classes (*F*_5,30_ = 3.04, *P* = 0.025; Fig. 2), treatment (*F*_2,58_ = 210.77, *P* < 0.001) and for the age class and treatment interaction (*F*_10,58_ = 3.91, *P* < 0.001). There was a significant difference among age classes within the in-air treatment, such that neonate pelt thermal conductivity in air was significantly higher than juvenile (*P* = 0.006) and subadult (*P* = 0.001) pelt conductivities in air. Pelt thermal conductivity was similar across age classes for the in-water treatment (*P* range: 0.9936–1.000) and for the oiled treatment (*P* range: 0.0732–1.000). Regression analysis revealed that sea otter thermal conductivity was significantly negatively correlated with hair density (*F*_1,40_ = 13.87, *r*^2^ = 0.2389, *P* = 0.0006044; Fig. 3), according to the equation *k* = −0.00004434 (hair density) - 0.1877.

Pelt thermal conductivities were significantly lower in water than in air across all age classes (*P* < 0.001; Fig. 2). Additionally, thermal conductivity of pelts in water was significantly lower than for oiled pelts across all age classes (*P* < 0.001). Subadult pelt thermal conductivity in air was higher than that for all other age classes in water (*P* range: < 0.0001–0.0483) except for adult pelt thermal conductivity in water (*P* = 0.0921). Juvenile pelts had a higher thermal conductivity in air than subadult (*P* = 0.006) and neonate (*P* = 0.001) pelt thermal conductivities in water. Adult, large pup, small pup, and neonate pelt thermal conductivities in air were significantly higher than that for all other age classes in water (*P* < 0.001 for all comparisons). Within each age class, in-air and oiled thermal conductivity values did not differ significantly (*P* range: 0.0647–1.000). Adult pelt thermal conductivity in air was significantly higher than large pup (*P* = 0.0425) oiled pelt thermal conductivity. Subadult and juvenile pelt thermal conductivities in air were significantly higher than adult (*P* = 0.013, *P* = 0.03), large pup (*P* < 0.001, *P* < 0.001), and small pup (*P* = 0.004, *P* = 0.012) oiled pelt thermal conductivities. Large pup, small pup, and neonate pelt thermal conductivities in air were not significantly different from any oiled pelt thermal conductivities across age classes (*P* range: 0.4662–1.000).

#### Pelt thickness

There was a significant interaction between age class and treatment for pelt thickness (*F*_10,64_ = 13.42, *P* < 0.001; Table 1). Pelt thickness differed significantly across age classes (*F*_5,33_ = 9.72, *P* < 0.001) and among treatments (*F*_2,64_ = 1252.65, *P* < 0.001; Table 1). Overall, pelt thickness decreased significantly when submerged and after oil application (*P* < 0.001 for all comparisons). Neonate and small pup pelts had significantly greater dry pelt thickness compared to all older age classes (*P* < 0.001 for all comparisons).

**Table 1:** Thicknesses (mm) of sea otter pelts (fur and skin) when dry, when submerged, and when treated with crude oil

**Age class**	**N dry**	**Dry pelt thickness (mm)**	**N submerged**	**Submerged pelt thickness (mm)**	**N oiled**	**Oiled pelt thickness (mm)**
Neonate	9	42.05 ± 2.66^a^	8	8.11 ± 2.17^c^	7	7.32 ± 1.12^c^
Small pup	5	43.12 ± 6.05^a^	5	9.12 ± 2.58^c^	5	8.24 ± 2.24^c^
Large pup	5	33.80 ± 6.14^b^	5	7.81 ± 2.66^c^	5	7.58 ± 1.30^c^
Juvenile	7	30.04 ± 2.38^b^	5	7.99 ± 1.54^c^	5	7.37 ± 1.39^c^
Subadult	9	28.85 ± 2.47^b^	7	8.88 ± 2.46^c^	8	8.55 ± 2.01^c^
Adult	9	30.86 ± 2.77^b^	5	9.33 ± 0.81^c^	5	8.16 ± 1.50^c^

Values (mean ± 1SD) are provided for each age class. N represents the number of individual pelt samples for each age class classified by treatment. Pelt thickness decreased significantly when wet and when oiled, compared with the dry pelt, for all age classes. Different superscript letters represent statistically significantly different means among age classes, within a treatment.

#### Thermal resistance

**Figure 4:**
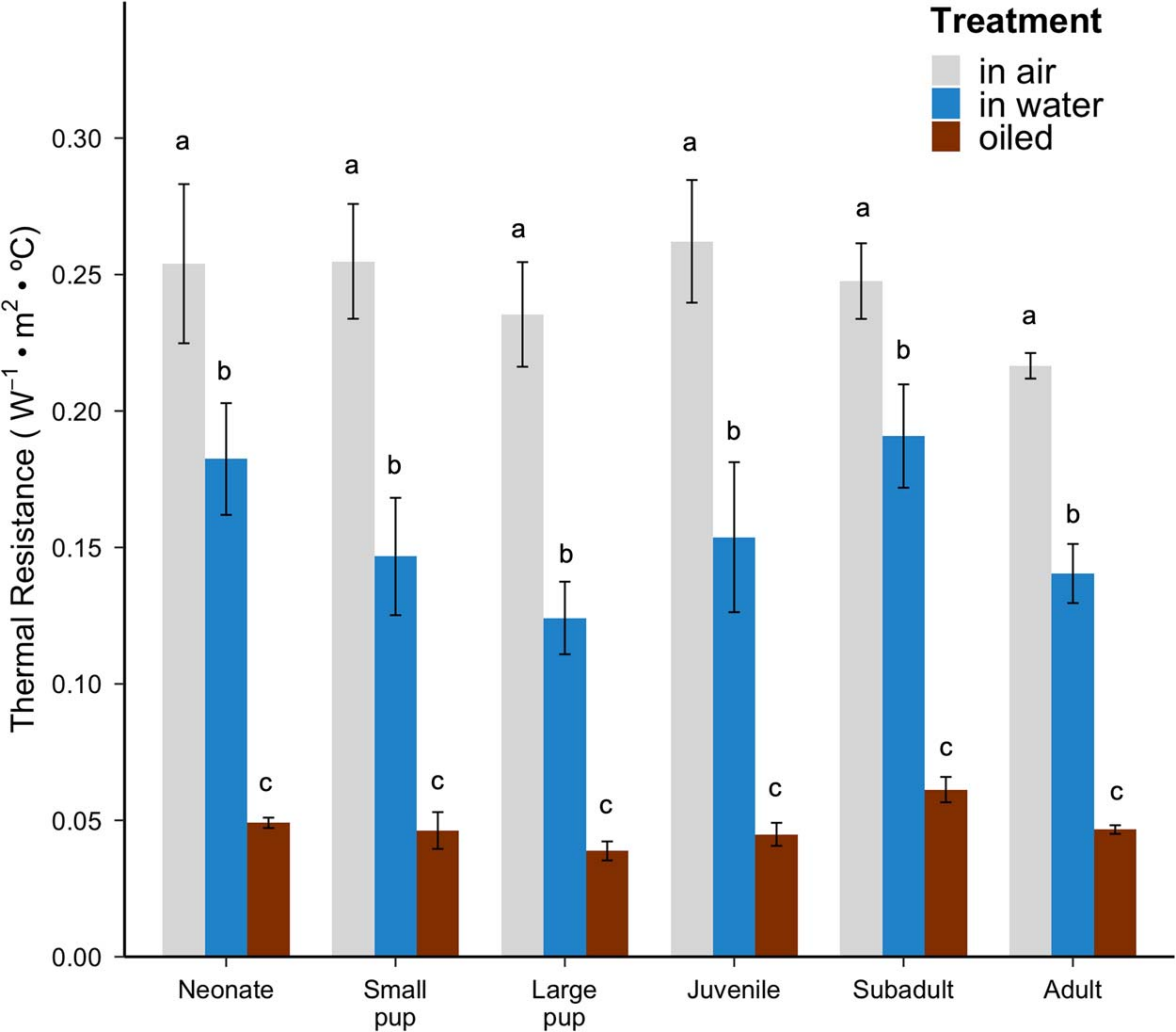
Thermal resistance (m^2^·°C·W^−1^) of sea otter pelts across ontogeny in air, in water and after oiling with crude oil. Heights of the bars and lines indicate means and standard errors for the associated treatment and age class: neonate (N = 9 in air, N = 8 in water, N = 7 oiled), small pup (N = 5 for all treatments), large pup (N = 5 for all treatments), juvenile (N = 6 in air, N = 5 in water, N = 5 oiled), subadult (N = 10 in air, N = 7 in water, N = 8 oiled) and adult (N = 9 in air, N = 5 in water, N = 5 oiled). Different letters above the bars indicate statistically significant findings among means.

Pelt thermal resistance was similar across age classes (*F*_5,35_ = 0.91, *P* = 0.487; Fig. 4), with no interaction detected between age class and treatment (*F*_10,61_ = 1.04, *P* = 0.423). However, pelt thermal resistance across treatments did differ significantly (*F*_2,61_ = 307.65, *P* < 0.001). Among all age classes, thermal resistance of pelts in air was significantly higher than that of pelts in water (*P* < 0.001) and that of oiled pelts (*P* < 0.001), and the thermal resistance of pelts in water was significantly higher than that of oiled pelts (*P* < 0.001).

#### Pelt cleaning

The amount of Dawn^®^ used to clean the pelts after the oiled treatment was similar across age classes (*F*_5,29_ = 0.544, *P* = 0.741; Table 2). However, the amount of time necessary to fully rid the pelts of crude oil did differ significantly across age classes (*F*_5,29_ = 3.482, *P* = 0.0138). The amount of cleaning time for neonate (*P* = 0.0181) and small pup (*P* = 0.0161) pelts was significantly less than the time required to clean subadult pelts (Table 2).

**Table 2:** The amount of Dawn^®^ (ml) required to remove crude oil from the pelts, and the amount of time (min) needed to clean the pelts

**Age class**	**N**	**Amount of Dawn** ^**®**^ **(ml)**	**Clean time (min)**
Neonate	7	9.86 ± 2.73	6.50 ± 1.89^a^
Small pup	5	11.40 ± 5.55	6.20 ± 1.10^a^
Large pup	5	12.80 ± 4.55	7.60 ± 0.89^ab^
Juvenile	5	10.60 ± 2.79	7.30 ± 0.57^ab^
Subadult	8	13.00 ± 5.13	9.06 ± 1.57^b^
Adult	5	12.40 ± 4.77	7.60 ± 1.64^ab^

Values (mean ± 1SD) are provided for pelt samples used in oiled thermal conductivity trials. N represents the number of individual pelt samples for each age class. Different superscript letters represent statistically significantly different means among age classes.

### Heat loss model

**Figure 5:**
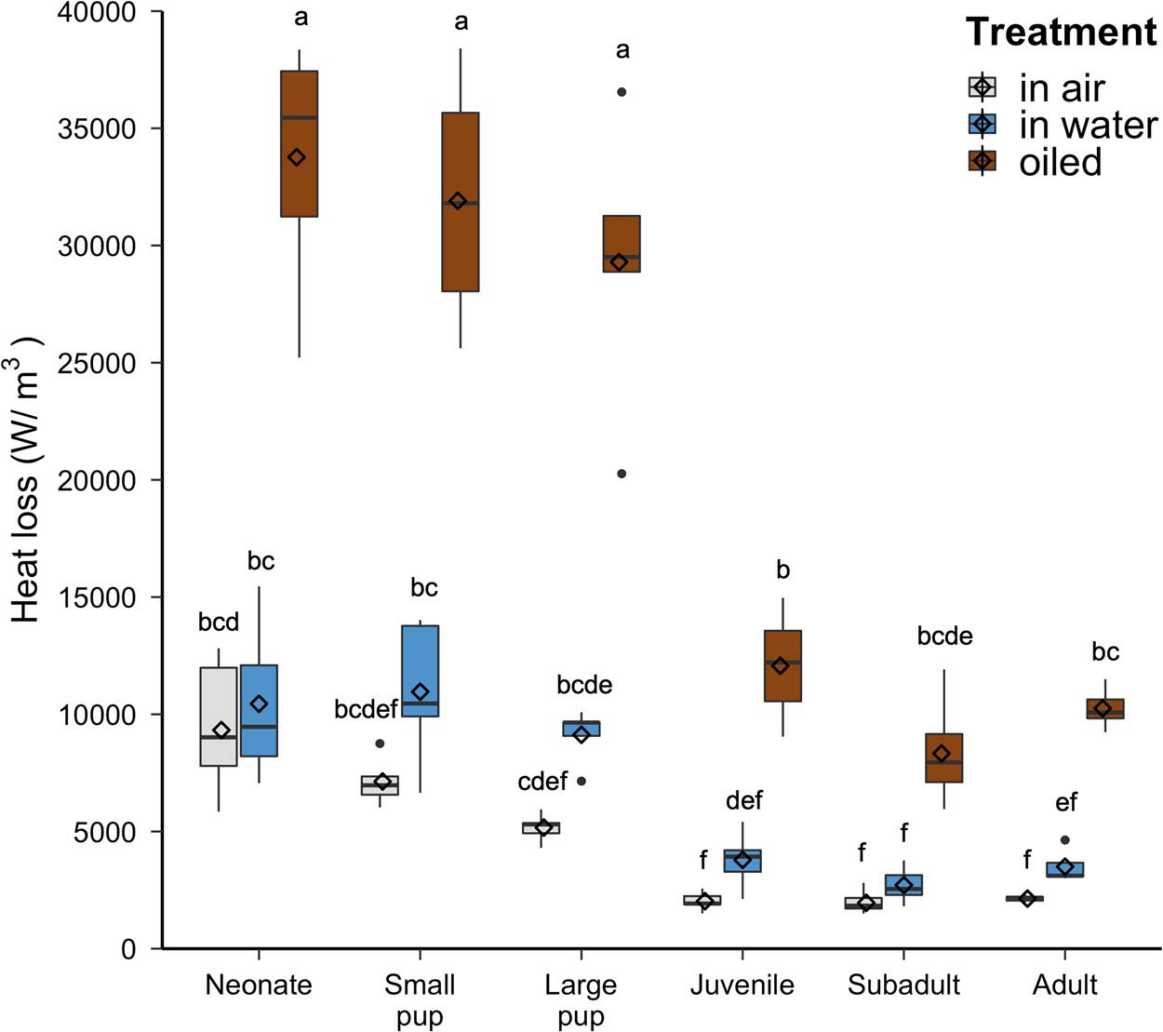
Heat loss per unit volume (W/m^3^) of sea otter pelts across ontogeny in air, in water and after oiling with crude oil. The horizontal line within each box indicates the median value and the box boundaries indicate the upper and lower interquartile range. Vertical lines indicate minimum and maximum values within 1.5 times the interquartile range. Individual points are outlier values >1.5 times and <3 times the interquartile range. Diamonds within boxplots indicate the mean values for each age class. Different letters above the boxes indicate statistically significant findings among means. Note that young sea otters are especially vulnerable to heat loss when oiled.

There were significant differences in the heat loss model across age class (*F*_5,30=_35.44, *P* < 0.001; Fig. 5), treatment (*F*_2,66_ = 424.07, *P* < 0.001), and for the age class and treatment interaction (*F*_*10*,66_ = 25.06, *P* < 0.001). Total heat loss was significantly higher when oiled than in air (*P* range: < 0.0001–0.0004) and in water (*P* range: < 0.0001–0.0140) across all age classes. Neonate, small pup, and large pup heat loss when oiled was significantly higher than that of all older age classes when oiled (*P* < 0.0001 for all comparisons). For both in-air and in-water estimates, neonates had significantly more heat loss than juveniles (*P* = 0.003, *P* = 0.0467), subadults (*P* < 0.0001, *P* < 0.0010), and adults (*P* < 0.0001, *P* = 0.0269). In water, neonates and small pups had significantly more heat loss than juveniles, subadults, and adults (*P* range: 0.0001–0.0.0091). Across all age classes, heat loss in air and in water was statistically similar (*P* range: 0.6157–1.0000).

## Discussion

### Thermal function of sea otter pelts

This study compared the insulative properties of southern sea otter pelage across ontogeny in three different treatments: in air, in water, and oiled. The higher conductivity of neonate pelts compared with juvenile and subadult pelts suggests that the natal pelage is a less efficient insulator in air compared with the pelts of older age classes (Fig. 2). This increased conductivity is due to the lower hair density of the natal pelage compared with adult pelage (Riordan *et al.*, 2023), as thermal conductivity is inversely related to hair density (Fig. 3). However, the greater loft of the natal pelage (Table 1) resulted in similar thermal resistance, and thus similar insulative effectiveness, across all age classes (Fig. 4). The longer guard hairs present in neonate and small pup pelts provide a thicker insulating layer of air in the fur. The fluffy nature of sea otter natal pelage makes the overall insulation equivalent to the adult pelage, even with a lower hair density.

Living in water is thermally challenging due to the higher thermal capacity and conductivity of water compared with air. Sea otter pelage plays an important role in insulation, helping to minimize contact between the cold ocean water and skin. Surprisingly, sea otter pelt thermal conductivity in fresh water was lower than in air across age classes (Fig. 2). Inside the thermal conductivity apparatus, the temperature-reading thermocouples were submerged and placed directly against the flattened air layer after cold water was applied. Sea otter fur maintains a stagnant layer of air, even when underwater, to prevent heat loss (Hammel, 1955; Ling, 1970; Kvadsheim and Aarseth, 2002). When sea otter fur is submerged, it becomes compressed and lies flat against the body (Table 1). The individual hairs overlap to prevent water penetration because the interstitial space between the underhairs decreases, and water has a high surface tension (Davis, 2019). Our results suggest that the intricate, interlocking nature of the underhairs is capable of trapping warm air at the base of the skin for prolonged periods if the air layer is not disturbed. When the pelts were submerged in water, the air layer was compressed but still maintained a similar thermal gradient across that smaller distance (16°C in air vs 13°C while submerged, on average); this resulted in lower pelt thermal conductivity values in water (Fig. 2).

Due to the lower hair density of the natal pelage, we originally predicted that sea otter pelts with the natal pelage would be less insulative compared with mature pelts. Instead, there was no difference in sea otter pelt thermal resistance in water across ontogeny. The difference in the pelt thickness for dry versus wet fur causes the fur to be less effective underwater (Fig. 4). The air layer is still trapping enough air to sustain the natural water-repelling qualities of the fur even under compression at the surface. Note, however, that the hydrostatic pressure imposed on the pelt during diving would cause the air layer to become further compressed, and at least some of the air would likely bubble out of the fur during the ascent of a dive (Liwanag *et al.*, 2012*a*; Nankey *et al.*, 2021). The compression at depth and the loss of air upon ascent would necessitate grooming to restore the air layer and the insulative properties of the pelt after a dive.

### Effects of oiling on sea otter pelts

The air layer of the fur must be preserved and well-groomed to minimize heat loss in water (Hammel, 1955). When fouled by crude oil or other petroleum products, sea otter fur suffers detrimental losses to its insulative properties. Previous studies found that oiling of sea otter fur causes a 70% decrease in thermal insulation, potentially leading to hypothermia (Davis *et al.*, 1988; Williams *et al.*, 1988). Our findings are consistent with previous research (Williams *et al.*, 1988) that found sea otter pelts have poorer thermal capabilities when oiled compared with intact pelts in air and in water (Fig. 4). We predicted that the oiled natal pelage would be less efficient at insulating compared with oiled mature pelts. The application of crude oil removed the air layer from the fur and caused a 5-fold reduction in the thermal resistance compared with pelts in air (Fig. 4), demonstrating that the presence of oil in the fur reduces the ability to maintain the air layer and the natural waterproofing feature of the pelt. This suggests that all sea otters may be vulnerable to the effects of oiling, regardless of age.

In addition, the amount of insulation provided by hairs varies with pelt thickness and hair density (Davis, 2019). Crude oil ruins the fur’s waterproofing and insulation abilities. In response, oiled northern sea otters (*Enhydra lutris kenyoni*) are more likely to haul out of the water to reduce body heat loss (Costa and Kooyman, 1982). In the event of an oil spill within the southern sea otter population range, oiled sea otters can become hypothermic in the water or if hauled out. To compensate for heat loss, sea otters will consequently need to increase metabolic rates (Siniff *et al.*, 1982). This is difficult because sea otters are already thermally compromised by their lower critical temperature of 20°C (Costa and Kooyman, 1982) and high thermal liability (Liwanag, 2010). The present study only focused on the external effects of oil to sea otter fur in a laboratory-based setting. Crude oil exposure can cause severe physiological issues to marine wildlife, and we did not investigate any internal toxicological impacts on sea otters.

Previous research has demonstrated the benefits of cleaning oiled sea otter pelts with Dawn^®^ dish soap (Williams *et al.*, 1988; Jessup *et al.*, 2012), and its use for washing oil contamination from wildlife has become standard practice for rehabilitators (Newman *et al.*, 2003; Jessup *et al.*, 2012; Tseng and Ziccardi, 2020). We found that a similar amount of Dawn^®^ allowed us to fully clean oil from natal pelage in less time than required to fully clean pelage of more mature animals (Table 2), likely due to the lower hair density of young sea otters (Riordan *et al.*, 2023). It is important to note that Dawn^®^ would also remove the natural oils that are important for fully restoring the waterproofing properties to the pelt. This requires sea otters in rehabilitation to naturally groom their fur to restore its natural oils and insulation (Jessup *et al.*, 2012).

### Scaling up: whole body insulation

Protection from heat loss is important for sea otters, as endotherms living in water. Water has a high thermal conductivity compared with air, and this creates a dynamic thermal challenge for sea otters to maintain a body temperature of 38.1°C (averaged from values in Morrison *et al.*, 1974; Costa and Kooyman, 1982; Davis *et al.*, 1988). It is especially difficult for smaller animals to maintain a body temperature greater than that of their environment, due to a high SA:V (Schmidt-Nielsen, 1990). Although our thermal resistance results were similar across age classes, these measurements in the laboratory are for pelt samples of the same surface area, and therefore do not account for differences in SA:V. By developing our heat loss model, we scaled our laboratory-based results to the whole animal (Fig. 1). Our findings based on this modeling have conservation implications, as they indicate younger sea otters are more vulnerable to the negative effects of oiling due to their large SA:V (Fig. 5). Previous research noted that sea otters transition from the natal pelage to a more adult-like pelage sometime between the small and large pup age classes (Riordan *et al.*, 2023). However, we still see that large pups are experiencing similar heat loss values to younger age classes (Fig. 5), even with pelage that resembles the adult pelage in morphology (Riordan *et al.*, 2023). Large pups are substantially smaller than sea otters in older age classes, and the higher SA:V of large pups increases their total heat loss. Thus, sea otter body size apparently contributes more to their overall rates of heat loss than the hair density of the pelt. Indeed, sea otters have a larger body size compared with their semi-aquatic and terrestrial relatives, reducing their SA:V and thus heat loss (Pabst *et al.*, 1999).

For adult sea otters, a smaller SA:V is beneficial because it decreases the amount of heat loss to the environment (Worthy and Edwards, 1990; Pabst *et al.*, 1999). Our thermal findings may explain why sea otter mothers keep small-bodied pups on their bellies and out of the water, because the fur is a better insulator when dry (Fig. 4). Past observations have noted that sea otter mothers carry their pups high on their chest, out of the water, while resting and swimming (Kenyon, 1969), and they spend a substantial amount of time grooming the pup’s fur (Sandegren *et al.*, 1973), potentially confirming the importance of keeping the pup’s fur as dry as possible.

### Implications

Despite the physiological challenges associated with the use of fur as the primary insulator in water, sea otters have remained successful at thermoregulation due to the morphology and thermal function of their unique pelage. The body size of sea otters plays an important role in the amount of heat loss to their environment, making young sea otters more prone to heat loss. Regardless of age class and pelage type, all sea otters are susceptible to negative anthropogenic events like oil spills that can cause detrimental fouling of the fur. This is because the maintenance of air trapped in the fur is critical for thermal insulation and survival. As a result of the infamous Exxon Valdez oil spill, sea otters experienced the highest direct mortality of any mammal, primarily due to their inability to effectively thermoregulate when oiled (Garrott *et al.*, 1993; Williams and Davis, 1995). Within the last 50 years, nine large-scale oils spills (≥5 574 000 gal) in the Pacific Ocean have affected the western US coastline (California Coastal Commission). Of those nine, eight oil spills occurred near the southern sea otter range, and multiple oil platforms remain in proximity to the population off Point Conception (Supplementary Fig. S1). Although all sea otters are vulnerable, pups are especially susceptible to mortality in the event of an oil spill due to their higher SA:V and overall rates of heat loss.

## Supplementary Material

Web_Material_coad095

## Data Availability

The data presented in this article are available in its online supplementary materials.
