# Supplementary material for: Effects of ontogeny and oiling on the thermal function of southern sea otter (Enhydra lutris nereis) fur
Source: Conserv Physiol. 2023 Dec 14;11(1):coad095. doi: 10.1093/conphys/coad095 (PMC10724463; doi:10.1093/conphys/coad095)
Supplement: Web_Material_coad095 [file web_material_coad095.zip › Heat_loss_equations.pdf]

Heat loss model equations

Head (sphere)

$$Head\ diameter = \frac{head\ girth}{\pi}$$

$$Head\ radius = \frac{head\ girth}{2\pi}$$

$$R_{hc} = head\ radius - \frac{3}{4}L_{water}$$

Axillary (cylinder)

$$Axillary\ radius = \frac{axillary\ girth}{2\pi}$$

$$R_{ac} = \ axillary\ radius - L_{water}$$

$$H_{cyl} = L_{body} - H_{cone} - head\ diameter$$

Tail (cone)

$$Tail\ radius = axillary\ radius \cdot 0.2714504$$

$$R_{tc} = tail\ radius - \frac{3}{4}L_{water}$$

Total head radius

$$R_{ht,air} = R_{hc} + \ \frac{3}{4}L_{air}$$

$$R_{ht,water} = R_{hc} + \ \frac{3}{4}L_{water}$$

$$R_{ht,oiled} = R_{hc} + \ \frac{3}{4}L_{oiled}$$

Total axillary radius

$$R_{at,air} = R_{ac} + \ L_{air}$$

$$R_{at,water} = R_{ac} + \ L_{water}$$

$$R_{at,oiled} = R_{ac} + \ L_{oiled}$$

Total tail radius

$$R_{tt,air} = R_{tc} + \ \frac{3}{4}L_{air}$$

$$R_{tt,water} = R_{tc} + \ \frac{3}{4}L_{water}$$

$$R_{tt,oiled} = R_{tc} + \ \frac{3}{4}L_{oiled}$$

Heat flux: sphere

$$Q_{sphere} = \frac{T_{MB} - T_A}{\frac{1}{4}\pi k_i \left[\frac{1}{R_{hc}} - \frac{1}{R_{ht}}\right]}$$

Heat flux: cylinder

$$Q_{cyl} = 2\pi \cdot H_{cyl} \cdot k_i \left[\frac{T_{MB} - T_A}{ln\left(\frac{R_{ac}}{R_{at}}\right)} \right]$$

Heat flux: cone

$$Q_{cone} = \left[\frac{\pi k_i (T_{MB} - T_A)}{H_{cone}} \right] (R_{tc} \cdot R_{tt})$$

Volumes

$$V_{sphere} = \frac{4}{3}\pi(R_{hc})^3$$

$$V_{cyl} = \pi(R_{ac})^2 \cdot H_{cyl}$$

$$V_{cone} = \frac{1}{3}\pi(R_{tc})^2 \cdot H_{cone}$$

Heat loss total

$$Heat_{tot,air} = \frac{Q_{sphere,air} + Q_{cyl,air} + Q_{cone,air}}{V_{sphere} + V_{cyl} + V_{cone}}$$

$$Heat_{tot,water} = \frac{Q_{sphere,water} + Q_{cyl,water} + Q_{cone,water}}{V_{sphere} + V_{cyl} + V_{cone}}$$

$$Heat_{tot,oiled} = \frac{Q_{sphere,oiled} + Q_{cyl,oiled} + Q_{cone,oiled}}{V_{sphere} + V_{cyl} + V_{cone}}$$

Abbreviation key

R_{hc}: head core radius (m)
R_{ac}: axillary core radius (m)
R_{tc}: tail core radius (m)
R_{ht}: total head radius (m)
R_{at}: total axillary radius (m)
R_{tt}: total tail radius (m)
k_i: thermal conductivity of the pelt for the specific treatment ($W \cdot m^{-1} \cdot °C^{-1}$)
L_i: pelt thickness for the specific treatment (m)
L_{body}: total body length (m)
H_{cone}: cone height (m) [tail length]
H_{cyl}: cylinder height (m) [trunk length]
Q_{sphere}: heat flux of a sphere (W)
Q_{cyl}: heat flux of a cylinder (W)
Q_{cone}: heat flux of a cone (W)

V_{sphere}: volume of sphere (m^3)
V_{cyl}: volume of cylinder (m^3)
V_{cone}: volume of cone (m^3)
T_{MB}: temperature at the muscle-skin interface (set to 36.5°C)
T_A: ambient temperature (set to 16°C for in air and 13°C for in water and oiled)
$Heat_{tot}$: total conductive heat transfer for the specific treatment (W/m^3)
