# Supplementary material for: Effects of ontogeny and oiling on the thermal function of southern sea otter (Enhydra lutris nereis) fur
Source: Conserv Physiol. 2023 Dec 14;11(1):coad095. doi: 10.1093/conphys/coad095 (PMC10724463; doi:10.1093/conphys/coad095)
Supplement: Web_Material_coad095 [file web_material_coad095.zip › R_code.pdf]

```
# packages used
library(tidyverse)
library(ggplot2)
library(lmerTest)
library(afex)
library(multcomp)
library(multcompView)
library(emmeans)

#################################
# thermal conductivity analysis #
#################################
df_k_R <- read.csv("k_R.csv", header=TRUE)
# thermal conductivity (k) linear mixed models
model_k_lmer <- lmer(k ~ 1
             + Age.Class * Treatment
             + (1|Sample.ID), data=df_k_R)
summary(model_k_lmer)
model_k_mixed <- mixed(k ~ 1
              + Age.Class * Treatment
              + (1|Sample.ID), data=df_k_R)
model_k_mixed
# connecting letters display for thermal conductivity
compare_k <- emmeans(model_k_lmer, pairwise ~ Age.Class*Treatment)
compare_k
k_letters <- cld(object = compare_k,
             adjust = "sidak",
             Letters = letters,
             alpha = 0.05)
k_letters


###############################
# thermal resistance analysis #
###############################
# thermal resistance (R) linear mixed models
model_R_lmer <- lmer(R ~ 1
             + Age.Class * Treatment
             + (1|Sample.ID), data=df_k_R)
summary(model_R_lmer)
model_R_mixed <- mixed(R ~ 1
              + Age.Class * Treatment
              + (1|Sample.ID), data=df_k_R)
model_R_mixed
```

```
###########################
# pelt thickness analysis #
###########################
df_pelt_thick <- read.csv("pelt_thickness.csv", header=TRUE)
# pelt thickness linear mixed model
model_pelt_thick <- lmer(thickness ~ 1
                   + Age.Class * Treatment
                   + (1|Sample.ID), data=df_pelt_thick)
summary(model_pelt_thick)
model_mixed_pelt_thick <- mixed(thickness ~ 1
                         + Age.Class * Treatment
                         + (1|Sample.ID), data=df_pelt_thick)
# connecting letters display for pelt thickness
compare_pelt_thick <- emmeans(model_pelt_thick, pairwise ~ Age.Class*Treatment)
compare_pelt_thick
pelt_thick_letters <- cld(object = compare_pelt_thick,
                adjust = "sidak",
                Letters = letters,
                alpha = 0.05)
pelt_thick_letters


##########################################
# amount of Dawn and clean time analysis #
##########################################
df_dawn_clean <- read.csv("Dawn_clean_time.csv",header=TRUE)
# amount of Dawn ANOVA
dawn_amt <- aov(Dawn.amt ~ Age.Class, data=df_dawn_clean)
summary(dawn_amt)
# clean time ANOVA
time_amt <- aov(Time.amt ~ Age.Class, data=df_dawn_clean)
summary(time_amt)
Tukey_time_amt <- TukeyHSD(time_amt)
# connecting letters display (cld) for clean time
Tukey_time_amt_cld <- multcompLetters4(time_amt, Tukey_time_amt)
print(Tukey_time_amt_cld)


################################################
# hair density & thermal conductivity analysis #
################################################
# hair density and thermal conductivity (k) linear regression
```

```
df_k_hair <- read.csv("k_hair_density.csv")
linear_regression_k_hair <- lm(k ~ Hair.density, data=df_k_hair)
linear_regression_k_hair
summary(linear_regression_k_hair)


#######################
# heat loss analysis #
######################
df_heat_loss <- read.csv("heat _loss.csv")
# heat loss linear mixed model
model_heatloss <- lmer(Heat.loss.total ~ 1
                  + Age.Class * Treatment
                  + (1|Sample.ID), data=df_heat_loss)
model_heatloss
model_heatloss_mixed <- mixed(Heat.loss.total ~ 1
                        + Age.Class * Treatment
                        + (1|Sample.ID), data=df_heat_loss)
model_heatloss_mixed
# connecting letters display for heat loss
compare_heatloss <- lsmeans(model_heatloss, pairwise ~ Age.Class*Treatment)
compare_heatloss
heatloss_letters <- cld(object = compare_heatloss,
                   adjust = "sidak",
                   Letters = letters,
                   alpha = 0.05)
heatloss_letters
```
