# Supplementary material for: Effects of ontogeny and oiling on the thermal function of southern sea otter (Enhydra lutris nereis) fur
Source: Conserv Physiol. 2023 Dec 14;11(1):coad095. doi: 10.1093/conphys/coad095 (PMC10724463; doi:10.1093/conphys/coad095)
Supplement: Web_Material_coad095 [file web_material_coad095.zip › Supplementary_figure_tables.pdf]

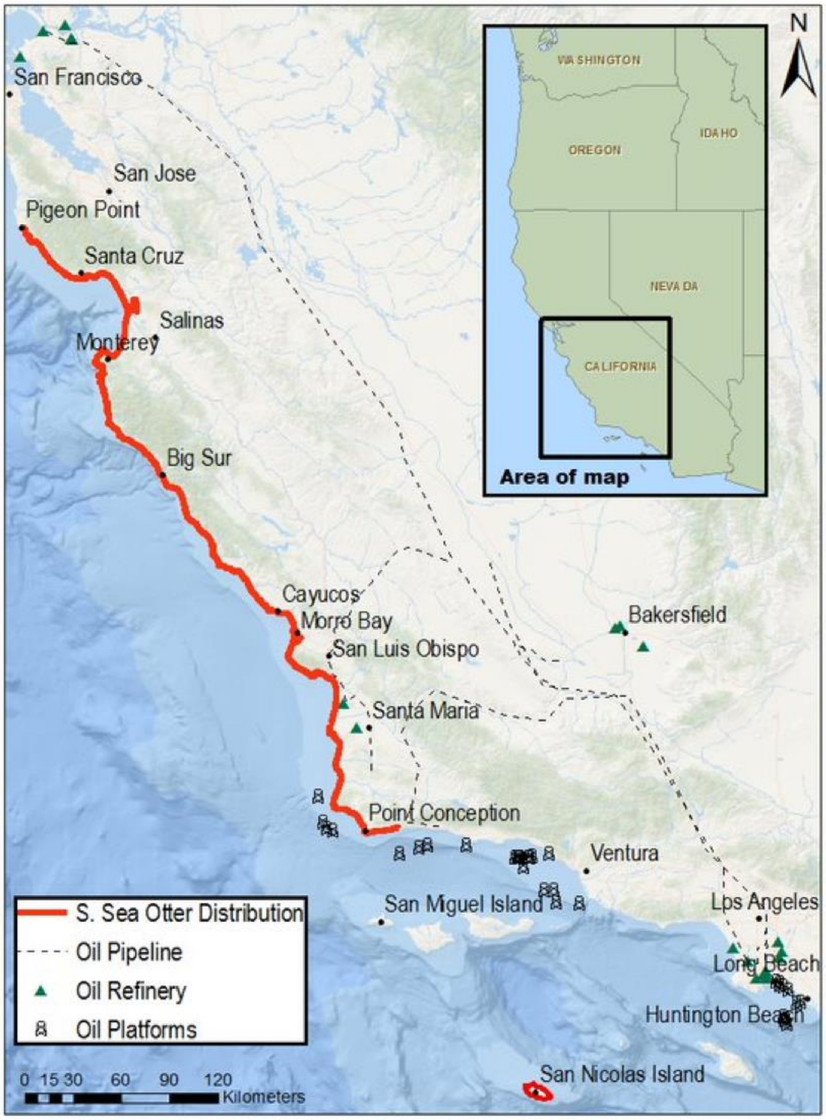

Figure S1. Site-scale map of the distribution of southern sea otter populations along the coast relative to crude oil pipelines, refineries, and platforms. California sea otter range data retrieved from USGS 2019 Population Census Results, Spring 2019 Data. Oil data received from the U.S. Energy Information Administration. Map Credit: Kate Riordan and Emily Levin (ArcMap).

Table S1. Model parameters used to estimate heat loss that were consistent across treatments. Mean values are provided for each age class.

**Age class**	**Body length (m) [L_{body}]**	**Head girth (m)**	**Head core radius (m) [R_{hc}]**	**Volume of sphere (m^3) [V_{sphere}]**	**Axillary girth (m)**	**Axillary core radius (m) [R_{ac}]**	**Cylinder height (m) [H_{cyl}]**	**Volume of cylinder (m^3) [V_{cyl}]**	**Tail girth (m)**	**Tail core radius (m) [R_{tc}]**	**Cone height (m) [H_{cone}]**	**Volume of cone (m^3) [V_{cone}]**
Neonate	0.56667	0.33554	0.04732	0.000445	0.26277	0.03371	0.30875	0.00110	0.07133	0.00527	0.15111	0.000005
Small pup	0.71920	0.34027	0.04732	0.000445	0.30553	0.03951	0.42809	0.00212	0.08294	0.00636	0.18280	0.000008
Large pup	0.81200	0.34500	0.04905	0.000496	0.37307	0.05156	0.49818	0.00417	0.10127	0.01026	0.20400	0.000023
Juvenile	0.85917	0.34973	0.04950	0.000509	0.68807	0.10152	0.53451	0.01731	0.18678	0.02373	0.21333	0.000126
Subadult	1.06333	0.35020	0.04907	0.000496	0.72760	0.10692	0.68624	0.02466	0.19751	0.02477	0.26563	0.000172
Adult	1.21333	0.35150	0.04895	0.000491	0.73809	0.10814	0.81723	0.03001	0.20036	0.02489	0.28422	0.000185

Table S2. Model parameters used to estimate heat loss that varied across treatments. Mean values are provided for each age class.

	Thermal conductivity (W·m-1·oC-1) [k_i]			Pelt thickness (m) [L_i]			Total head radius (m) [R_{ht}]			Total axillary radius (m) [R_{at}]			Total tail radius (m) [R_{tt}]			Total heat loss (W/m³) [$Heat_{tot}$]		
**Age class**	**in air**	**in water**	**oiled**	**dry**	**wet**	**oiled**	**in air**	**in water**	**oiled**	**in air**	**in water**	**oiled**	**in air**	**in water**	**oiled**	**in air**	**in water**	**oiled**
Neonate	0.1792	0.0466	0.1466	0.0315	0.0061	0.0054	0.0789	0.0534	0.0526	0.0758	0.0418	0.0434	0.0368	0.0114	0.0106	9325.19	10447.97	33768.39
Small pup	0.1714	0.0660	0.1845	0.0323	0.0068	0.0062	0.0796	0.0542	0.0535	0.0826	0.0486	0.0477	0.0387	0.0132	0.0125	7132.97	10962.95	34250.93
Large pup	0.1458	0.0620	0.1985	0.0254	0.0059	0.0057	0.0744	0.0549	0.0547	0.0854	0.0594	0.0591	0.0356	0.0161	0.0159	5166.63	9125.80	29291.91
Juvenile	0.1176	0.0560	0.1665	0.0225	0.0060	0.0055	0.0720	0.0557	0.0552	0.1316	0.1095	0.1089	0.0463	0.0297	0.0293	2026.58	3789.05	12069.59
Subadult	0.1198	0.0483	0.1444	0.0216	0.0067	0.0064	0.0707	0.0557	0.0555	0.1358	0.1158	0.1151	0.0464	0.0314	0.0312	1956.21	2711.81	8329.07
Adult	0.1428	0.0676	0.1772	0.0231	0.0070	0.0061	0.0721	0.0559	0.0551	0.1390	0.1175	0.1163	0.0310	0.0319	0.0310	2135.80	3499.96	10254.55
